# β-Hydroxybutyrate Elicits Favorable Mitochondrial Changes in Skeletal Muscle

**DOI:** 10.3390/ijms19082247

**Published:** 2018-08-01

**Authors:** Brian A. Parker, Chase M. Walton, Sheryl T. Carr, Jacob L. Andrus, Eric C. K. Cheung, Michael J. Duplisea, Esther K. Wilson, Carrie Draney, Daniel R. Lathen, Kyle B. Kenner, David M. Thomson, Jeffery S. Tessem, Benjamin T. Bikman

**Affiliations:** 1 Department of Physiology and Developmental Biology, Brigham Young University, Provo, UT 84604, USA; info@youarelimitless.org (B.A.P.); chase.m.walton@gmail.com (C.M.W.); sherylteresa@gmail.com (S.T.C.); jacob.l.andrus@gmail.com (J.L.A.); eric.cheung.801@gmail.com (E.C.K.C.); mduplisea@hotmail.com (M.J.D.); olsenkezia@gmail.com (E.K.W.); david_thomson@byu.edu (D.M.T.); 2 Department of Nutrition, Dietetics and Food Science, Brigham Young University, Provo, UT 84604, USA; carriedraney@gmail.com (C.D.); drlathen@gmail.com (D.R.L.); kyle.kener@gmail.com (K.B.K.); jeffery_tessem@byu.edu (J.S.T.)

**Keywords:** ketones, β-hydroxybutyrate, mitochondria, ceramides

## Abstract

The clinical benefit of ketosis has historically and almost exclusively centered on neurological conditions, lending insight into how ketones alter mitochondrial function in neurons. However, there is a gap in our understanding of how ketones influence mitochondria within skeletal muscle cells. The purpose of this study was to elucidate the specific effects of β-hydroxybutyrate (β-HB) on muscle cell mitochondrial physiology. In addition to increased cell viability, murine myotubes displayed beneficial mitochondrial changes evident in reduced H_2_O_2_ emission and less mitochondrial fission, which may be a result of a β-HB-induced reduction in ceramides. Furthermore, muscle from rats in sustained ketosis similarly produced less H_2_O_2_ despite an increase in mitochondrial respiration and no apparent change in mitochondrial quantity. In sum, these results indicate a general improvement in muscle cell mitochondrial function when β-HB is provided as a fuel.

## 1. Introduction

Once considered “metabolic garbage,” ketones have become the focus of significant efforts within the realm of cardiometabolic research. Recent discoveries have revealed that ketones, such as acetoacetate and its precursor β-hydroxybutyrate (β-HB), are not only viable fuel sources for all cells with mitochondria, including the brain [1] but are also legitimate signaling molecules, eliciting advantageous changes in inflammation [2], cognition [3,4], oxidative stress [5] and more. Beyond pathology, ketones may also be a relevant metabolic fuel in the context of physical activity, improving athletic performance [6] and myocardial adenosine triphosphate (ATP) generation [7]. Relevant, if not central, to each of these instances is the degree to which the ketones alter mitochondrial function.

Unsurprisingly, mitochondria are responsive to ketones, as it is converted to acetyl-Coa and catabolized within the mitochondrial matrix. However, glucose and fatty acid oxidation notably utilizes the same oxidative processes (i.e., via acetyl-Coa conversion) without eliciting the same numerous cellular benefits noted above. Thus, simple oxidation of the ketone fails to explain the disparate effects of these nutrients on organismal function.

Skeletal muscle represents the largest insulin-responsive tissue in the body and a defect in insulin signaling in muscle critically contributes to the mounting glycemic burden evident with prolonged insulin resistance, eventually resulting in frank type 2 diabetes. Disrupted mitochondrial function is thought to at least contribute to muscle insulin resistance [8,9], likely due to increased oxidative stress and altered insulin signaling [10,11,12], both of which may be affected by changes in mitochondrial morphology [8].

To a degree, previous findings of beneficial mitochondrial adaptations to ketones may offer insight into the benefits of ketosis, a state of mildly elevated blood ketones. Whether through the use of a ketogenic diet or the consumption of exogenous ketones, limited evidence suggests a generally favorable metabolic milieu [13,14,15,16]. To this end, the purpose of this study was to elucidate the effects of β-HB on myriad aspects of skeletal muscle mitochondrial physiology, including respiration, ATP and H_2_O_2_ emission and morphology.

## 2. Results

### 2.1. β-Hydroxybutyrate Elicits Favorable Changes in Mitochondrial Respiration in Muscle Cells

**Figure 1:**
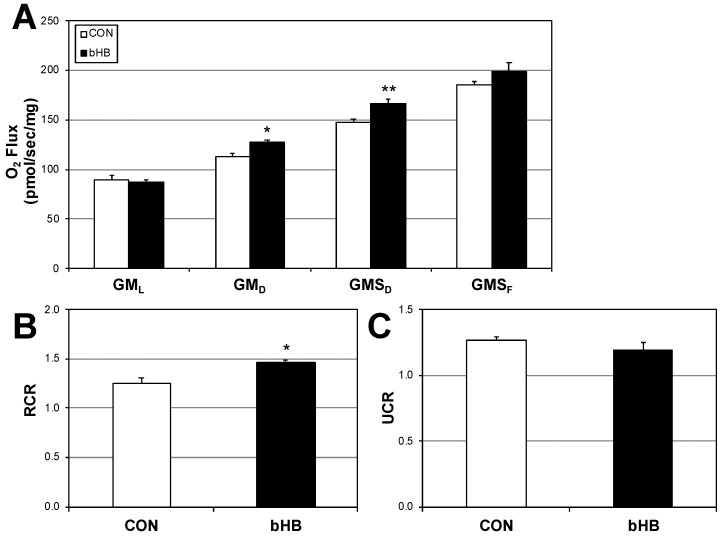

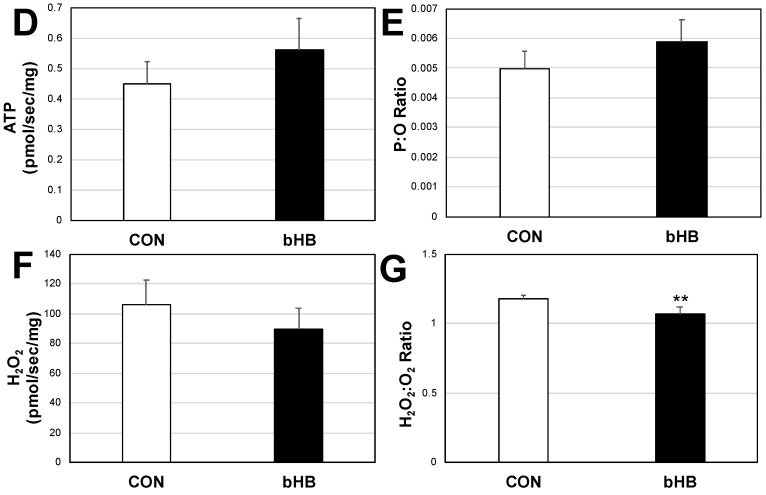
β-Hydroxybutyrate favorably alters mitochondrial function. C2C12 myotubes were treated with PBS (CON) or with β-Hydroxybutyrate (β-HB; 5 mM) for 24 h. To measure mitochondrial respiration (**A**), cells were treated with GM*_L_*: glutamate (10 mM) + malate (2 mM); GM*_D_*: + ADP (2.5 mM); GMS*_D_*: + succinate (10 mM); GMS*_F_*: + FCCP (0.05 μM). Respiratory control ratio ((**B**); RCR; GM*_D_*/GM*_L_*) and uncoupling control ratio ((**C**); UCR; GMS*_F_*/GMS*_D_*) were determined from the respiration assay. ATP production (**D**), ATP:O_2_ ratio (P:O; (**E**), H_2_O_2_ production (**F**) and H_2_O_2_:O_2_ ratio (**G**) were determined by measuring fluorescence. * *p* < 0.05; ** *p* < 0.01; *n =* 6.

Following 24 h of treatment with β-hydroxybutyrate β-HB), C2C12 myotubes had slight, yet significant elevations in mitochondrial respiration rate (Figure 1A), including after the addition of ADP (GMD) that continued with addition of succinate (GMSD) but not with carbonyl cyanide-4-(trifluoromethoxy)phenylhydrazone (FCCP). These changes were further evident in a significant elevation in respiratory control ratio (RCR), a general indicator of mitochondrial “fitness” (Figure 1B). Uncoupling control ratio (UCR; Figure 1C) was similar across the treatments.

Analysis of mitochondrial respiration (Figure 1A–C) simply indicates the rate of oxygen use. Thus, we sought to better understand the functional effect of this altered oxygen use by measuring ATP production and H_2_O_2_ emission. Firstly, we found that ATP production was similar across the treatments—β-HB addition had no significant effect, despite an upward trend (Figure 1D,E). However, H_2_O_2_ emission (Figure 1F) tended to change, significantly diminishing when compared with O_2_ consumption (Figure 1G).

### 2.2. β-Hydroxybutyrate Increases Cell Viability

**Figure 2:**
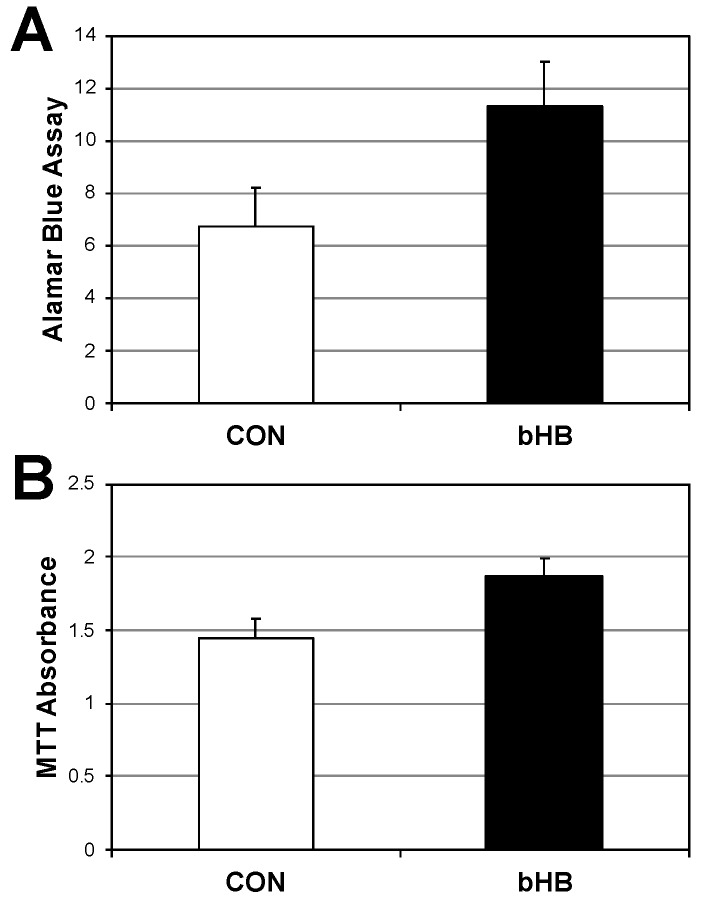
β-Hydroxybutyrate increases myotube viability. Following treatment with PBS (CON) or β-Hydroxybutyrate (β-HB; 5 mM) for 24 h, myotubes were measured for viability via Alamar Blue (**A**) or with MTT (**B**) to determine metabolic activity. *p* < 0.05; *n* = 6.

Given that exposure of C2C12 cells to β-HB enhances mitochondrial respiration, we sought to determine if this exposure increases cellular viability. C2C12 cells were cultured in the presence of vehicle or β-HB for 48 h. Cellular viability was measured using the Alamar Blue and proliferation via MTT assay (Figure 2A,B, respectively). C2C12 cells cultured in the presence of β-HB had increased cell viability and growth.

### 2.3. β-Hydroxybutyrate Induces Mitochondrial Fusion

**Figure 3:**
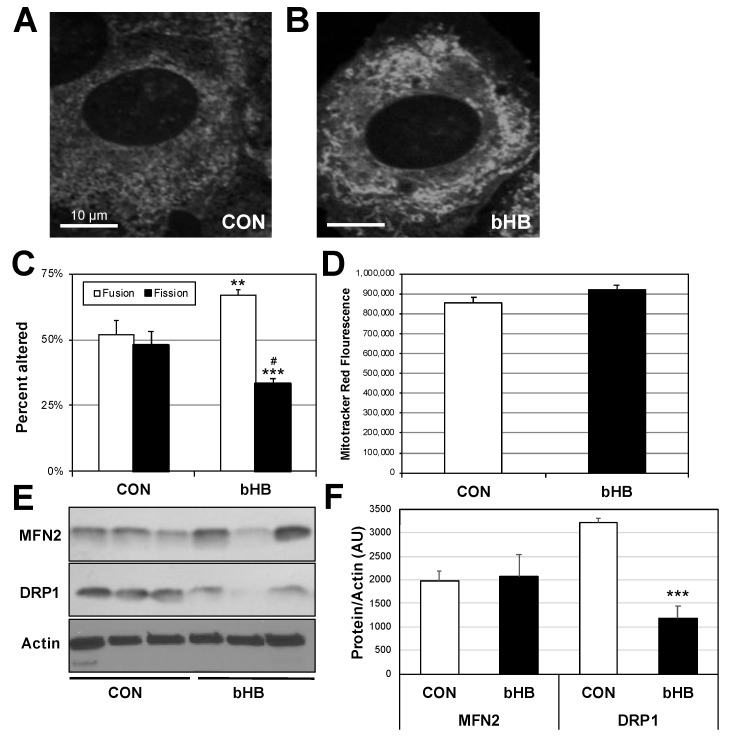
β-Hydroxybutyrate increases mitochondrial content and function. C2C12 myotubes were treated with vehicle (PBS; CON) or with β-Hydroxybutyrate (β-HB; 5 mM) for 24 h then incubated with MitoTracker and florescence was imaged to determine morphology (**A**–**C**) and quantified by plate reader assay ((**D**); *n* = 6). In parallel, cells were used to detect mitofusin-2 (MFN2) and dynamin-related peptide-1 (DRP1) protein expression ((**E**,**F**); *n* = 3). ** *p* < 0.01; *** *p* < 0.001 for β-HB versus CON; # *p* < 0.05 for fission vs. fusion.

We previously found that mitochondrial morphology alters mitochondrial physiology, including respiration and reactive oxygen species production [8]. In an effort to better understand a potential non-energetic mechanism whereby β-HB alters mitochondrial respiration, we scrutinized mitochondrial morphology and amount in C2C12 myotubes following β-HB treatment. There were no detectable differences in mitochondrial amount between treatments (Figure 3D). Whereas the control cells had a generally even degree of mitochondria that exhibited contact or no contact with adjacent mitochondria (Figure 3A), which we defined as fusion or fission, respectively, the β-HB-treated cells exhibited a shift in increased mitochondrial fusion (Figure 3B,C). These morphological differences were supported by disparate levels of mitochondrial proteins that mediate fusion and fission. While β-HB treatment elicited no significant effect on MFN2, which promotes fusion, it did significantly reduce DRP1, a key protein known to mediate mitochondrial fission (Figure 3D,E).

### 2.4. Myotube Ceramides Are Reduced with β-HB Treatment

**Figure 4:**
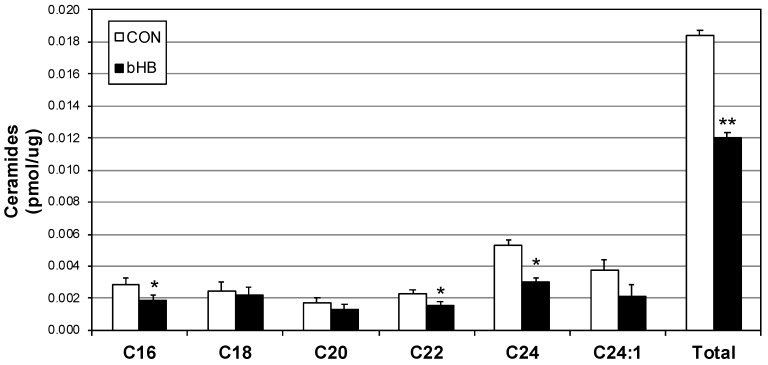
β-Hydroxybutyrate reduces select ceramide species. Following treatment with vehicle (PBS; CON) or with β-Hydroxybutyrate (β-HB; 5 mM) for 24 h, lipids were isolated from C2C12 myotubes then analyzed via lipidomics (*n* = 6). * *p* < 0.05; ** *p* < 0.01 for β-HB versus CON.

Despite being necessary to cellular health, ceramides mediate multiple pathological cellular processes [17,18,19], including forcing sustained mitochondrial fission [8]. Due to our findings of reduced mitochondrial fission in myotubes following β-HB treatment, we probed ceramide levels and found a small but significant reduction in multiple ceramide species, including C16, C22 and C24 (Figure 4).

### 2.5. Ketogenic Diet in Rats Alters Muscle Mitochondrial Physiology

**Figure 5:**
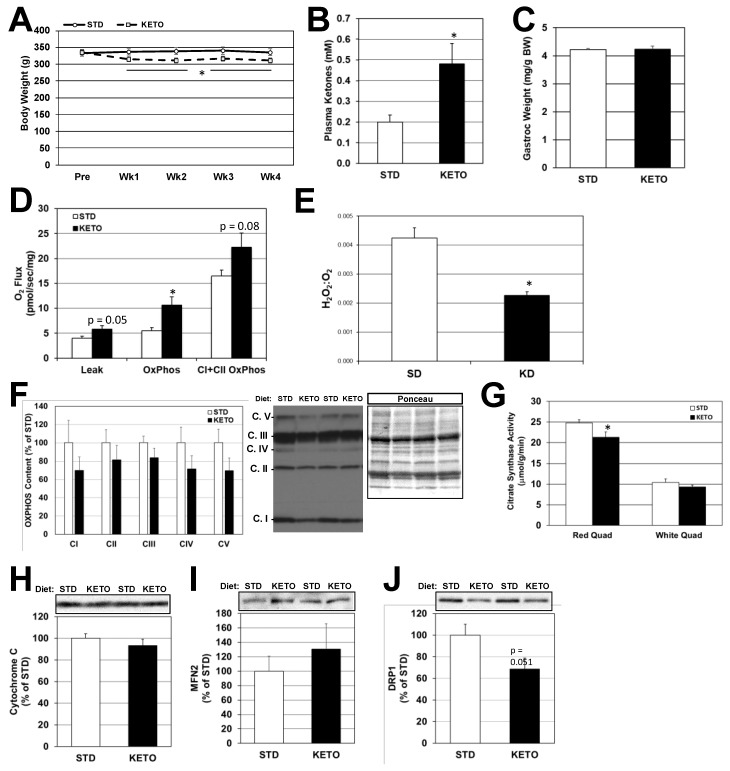
Ketogenic diet induces weight loss and enhances mitochondrial respiration without increased mitochondrial content in skeletal muscle from rats. Fisher 344 rats were pair-fed either a standard (STD) or ketogenic (KETO) diet for 4 weeks. Body weight (**A**) was measured weekly. At the end of the treatment period, blood and skeletal muscle were removed from the rats. Plasma β-hydroxybutyrate (**B**) and gastrocnemius (Gastroc) weight (**C**) were measured. Mitochondrial respiration (**D**) and H_2_O_2_:O_2_ ratio (**E**) were determined as in Figure 1. Components of the oxidative phosphorylation system (**F**), citrate synthase activity (**G**) and cytochrome C content (**H**) were measured as markers of mitochondrial content. Protein expression for mitofusin-2 ((**I**); MFN2) and dynamin-related peptide-1 ((**J**); DRP1) protein expression were assessed as markers of mitochondrial fusion vs. fission. * *p* < 0.05; *n* = 6.

Rats on a ketogenic diet (KETO) diet were pair-fed based on the consumption of standard (STD)-fed rats, such that caloric intake was not different between diets. Within 1 week on the KETO diet, body weight was significantly reduced by 6–8% compared to the STD-fed group (Figure 5A) and this persisted through the 4-week experimental period. After 3.5 weeks of feeding, plasma ketones were 140% higher in the KETO- versus STD-fed rats (Figure 5B; 0.49 vs. 0.2 mM, respectively). Muscle mass was not affected by diet (Figure 5C). Mitochondrial respiration was slightly elevated in the red quadriceps (Figure 5D), while H_2_O_2_ to O_2_ ratio was lower in muscle from KETO-fed rats (Figure 5E). Components of the oxidative phosphorylation complexes were not significantly different in KETO versus STD-fed groups, though they tended to be lower in the KETO-fed rats (Figure 5F,H). Citrate synthase activity, on the other hand, was significantly lower in the red quadriceps muscles (but not white quadriceps) of KETO versus STD-fed rats (Figure 5G). MFN2 and DRP1 levels were also not significantly affected by diet (Figure 5I,J), although a very strong trend (*p* = 0.051) for decreased DRP1 levels in the KETO-fed muscle was observed.

## 3. Discussion

The purpose of this study was to establish an underlying understanding of the actions of ketones on skeletal muscle cell physiology and mitochondrial function. We found that β-hydroxybutyrate (β-HB) increased mitochondrial respiration, with no loss of ATP production, yet a significant reduction in H_2_O_2_ emission. Furthermore, β-HB enhanced muscle viability, promoting survival in the wake of a noxious stimulus. Lastly, β-HB promoted a general shift towards mitochondrial fusion, which may be a result of reduced ceramides. Altogether, these results suggest a favorable influence of β-HB on muscle cell function.

Current dietary patterns, including macronutrient composition and meal frequency, result in very few instances of ketosis due to consistent elevations in insulin, which inhibits ketogenesis. However, ketosis, a stated of mildly elevated ketones, can be achieved through either dietary intervention (i.e., fasting or low-carbohydrate diet) or the consumption of exogenous ketones. While neither intervention has been studied in the context of understanding ketone-induced muscle cell mitochondrial physiology to the extent outlined in our study, there are nevertheless clues that suggests enhanced mitochondrial function in muscle.

The bulk of research exploring the mitochondrial effects of ketones use neurons as the cell of interest, which is not surprising given the pronounced neurological benefits of ketones, including reduced seizures [20], migraines [21] and improved cognition in both states of compromised [22] and healthy [23] neurological function. Some of these reported mitochondrial benefits of ketones in neurons are a reduction in oxidative stress and enhanced survival [24], similar to what we found in muscle cells in the studies herein. Despite this neuron-heavy focus, some previous evidence exists that suggests a mitochondrial benefit in muscle, albeit indirectly. Ahola-Erkkila et al. [25], in studying a model of mitochondrial myopathy, found that β-HB helped maintain mitochondrial respiration and morphology within muscle tissue, potentially slowing muscle loss with myopathies. This, combined with our observation of enhanced β-HB-induced myotube viability, presents a provocative paradigm with states of ketosis—that ketones promote muscle cell survival. Thus, perhaps ketones, in addition to serving other roles, act to preserve muscle mass in insulin-reduced or -deficient states, such as untreated type 1 diabetes, fasting, or a ketogenic diet. Beyond skeletal muscle, it is tempting to speculate that ketones may also alter cardiac muscle viability. Aubert et al. [26] recently found that heart failure due to hypertrophic cardiomyopathy is typified by a fuel shift, wherein cardiomyocytes rely more heavily on ketone catabolism. Moreover, Sato et al. [7] observed an increased ATP efficiency in myocardium fueled with ketones compared with other fuel sources.

Our findings of increased muscle cell viability when incubated with ketones may explain observations of maintained, or even increased, muscle mass in humans when fed an isocaloric, low-carbohydrate ketogenic diet. Specifically, Volek et al. [27] found that lean body mass increased roughly 1 kg in six weeks compared with control-fed subjects, which may stem from a ketone-induced muscle protein sparing effect [6,28,29]. Regardless of whether ketones induce a muscle-sparing effect via enhanced muscle cell viability, ketosis does not appear to hinder muscle function. Our findings of maintained ATP production, reduced H_2_O_2_ emission and increased muscle cell viability may provide molecular insight into how muscle performance is enhanced, or at least not compromised, when relying more heavily on ketones as ketone availability is increased. Ketone-induced enhanced muscle cell viability may not only reveal a novel mechanism whereby muscle is able to resist damage following intense physical activity but also perhaps in explicit muscle pathologies, such as muscle dystrophy [30].

Much of the mitochondrial and viability improvements in muscle cells could be explained by the β-HB-induced reduction in ceramides. Ceramides mediate deleterious changes in both mitochondrial function [8] and nutrient transport [31]. Moreover, the reduction in ketones may elicit improved viability due to a reduction in ceramide-mediated apoptotic signaling [17].

To determine the effect of nutritional ketosis on skeletal muscle in vivo, rats were fed a very low carbohydrate ketogenic diet for 4 weeks. Despite equal caloric intake, the KETO-fed rats weighed significantly less after 1 week on KETO versus STD-fed controls. A loss of muscle mass did not account for this weight loss; diet did not affect gastrocnemius weight. It may be due, at least in part, to a loss of glycogen and associated water from the liver and muscle, though this was not measured in the current study and evidence in humans suggests muscle glycogen is comparable in low-carbohydrate-fed to high-carbohydrate-fed individuals [32].

We observed decreased maximal citrate synthase activity in red but not white, muscle from KETO versus STD-fed rats, which may indicate reduced total mitochondrial number. This is consistent with previous work [33] in which similar findings were found in the mixed gastrocnemius of rats fed a ketogenic diet for 8 weeks. Despite the reduction in citrate synthase activity, red quadriceps muscles from KETO-fed rats had increased respiration, perhaps evidence of a compensatory mechanism. Moreover, consistent with our findings in βHB-treated myotubes, KETO feeding also resulted in a very nearly significant (*p* = 0.051) reduction in DRP1 protein content, suggesting that nutritional ketosis protects muscle against mitochondrial fragmentation. This effect is likely specific to an isocaloric ketogenic diet versus ad libitum high fat (60%) diet, which was reported to increase DRP1 in young rats [34] and may explain the improved H_2_O_2_ emission, something we’ve reported previously [8]. Our observed changes in DRP1 tempt the conclusion that KETO alters ceramides in whole muscle tissue [8]. However, the lack of ceramide analysis from the in vivo studies prevents such a conclusion and is an obvious limitation. We expect to report on the specific ketone-ceramide relationship in whole tissue in the near future.

In sum, the results of this study lend support for the growing scientific reevaluation of ketones as viable, even beneficial, metabolic signals. Moreover, our findings elucidate a potential mechanism whereby ketones elicit a protective effect on muscle cells and help maintain an overall favorable metabolic milieu. Future studies from human muscle tissue in states of ketosis are necessary to establish the relevance of these findings.

## 4. Materials and Methods

### 4.1. Cell Culture

C2C12 murine myoblast cells were maintained in DMEM (Dulbecco’s modified Eagle’s medium; D6546, Sigma-Aldrich, Saint Louis, MO, USA) plus 10% FBS (Invitrogen, Carlsbad, CA, USA). For differentiation into myotubes, C2C12 myoblasts were grown to confluency and the medium was replaced with DMEM plus 10% horse serum (Invitrogen). Myotubes were used for experiments on day 4 of differentiation. For β-hydroxybutyrate treatments (β-HB), cells were incubated with β-HB (54965, Sigma-Adrich) for 24 h at 5 mM.

### 4.2. Animals

All animal procedures were approved (18-0101, 9 February 2018) by the institutional animal care and use committee at Brigham Young University. 5-month-old male Fisher 344 rats (*n* = 6/group) were acclimatized for 1 week after arrival at the animal facility and were then pair-fed with either standard diet (STD; Envigo Teklad Rodent Diet, 8604; 32% protein, 14% fat, 54% carbohydrate) or ketogenic diet (KETO; Envigo Teklad custom diet, TD.10911; 22.4% protein, 77.1% fat, 0.5% carbohydrate) for 4 weeks. Each day the chow remaining in the cage was weighed and replaced with fresh chow. Body weight for the rats was recorded weekly. At 4 weeks, the rats were euthanized and blood, gastrocnemius and quadriceps muscles were removed. Plasma ketone levels were measured using the Abbott Precision Xtra Ketone Monitoring System. White and red quadriceps muscle was separated and a small sample of the red quadriceps (~10 mg) was prepared for assessment of mitochondrial respiration as described above. The remaining muscle was frozen at the temperature of liquid nitrogen and stored at −90 °C for later analysis.

### 4.3. Cell Viability Assays

Cells were plated at a concentration of 2 × 10^5^ cells/mL in 24-well plates (at 1 mL/well) or in 96-well plates (at 100 μL/well), then cultured for 48 h total with vehicle (water; CON) or 5 mM β-HB (β-HB). Cellular proliferation was determined by MTT assays (Sigma-Aldrich) and Alamar Blue assays (Sigma-Aldrich). Absorbance for the MTT and Alamar Blue assays were determined on a BioTek Synergy 2 plate reader.

### 4.4. Mitochondrial Morphology

C2C12 myoblasts were grown to confluence in chamber slides (NUNC Lab-Tek II Chambered Coverglass System; 155382) and differentiated at day 4. The mitochondrial dye MitoTracker Red CMXRos (Molecular Probes, Eugene, OR, USA; M7512), dissolved in anhydrous DMSO, was added to cultured myotubes at a concentration of 250 nM. The cells were incubated for 30 min at 37 °C in the dark and then visualized using a confocal microscope (Olympus IX81, Center Valley, PA, USA). Following image capture, a blind assessment of mitochondrial morphology was performed and quantified.

### 4.5. Mitochondrial Respiration

Cells and tissue were prepared for mitochondrial respiration as described previously [35,36,37,38] before being transferred to respirometer chambers using the Oroboros O2K oxygraph (Oroboros, Innsbruck, Austria). Electron flow through complex I was supported by glutamate + malate (10 mM and 2 mM, respectively) to determine leak oxygen consumption (GM*_L_*). Following stabilization, adenosine diphosphate (ADP) (2.5 mM) was added to determine oxidative phosphorylation capacity (GM*_D_*). Succinate was added (GMS*_D_*) for complex I + II electron flow into the Q-junction. To determine full electron transport system capacity in cells over oxidative phosphorylation, the chemical uncoupler carbonyl cyanide 4-(trifluoromethoxy) phenylhydrazone (FCCP) was added (0.05 μM, followed by 0.025 μM steps until maximal O_2_ flux was reached). Lastly, residual oxygen consumption was measured by adding antimycin A (2.5 μM) to block complex III action, effectively stopping any electron flow, which provides a baseline rate of respiration. Following respiration protocol, samples were removed from the chambers and used for further analysis, including protein quantification.

### 4.6. ATP Analysis

Following the culture period, cells were transferred to 2.5 mM glucose in SAB buffer for 2 h, followed by transfer to either 2.5 mM glucose SAB buffer or 16.7 mM glucose SAB buffer for 1 h. Cells were washed with PBS, harvested by trypsinisation and pelleted by centrifugation. The cells were lysed in 150 μL 1M perchloric acid on ice to precipitate cellular proteins. Lysate was centrifuged at 20,000× *g* for 10 min, after which 150 μL supernatant was transferred to a new tube with 150 μL 1M KOH. ATP was quantified using the ATP assay kit (Life Technologies, Carlsbad, CA, USA).

### 4.7. H_2_O_2_ Emission

H_2_O_2_ emission was measured using an Amplex Red Hydrogen Peroxide/Peroxidase Assay kit (Molecular Probes; A22188) as described previously [8]. A reaction mixture containing 50 μM Amplex Red and 0.1 unit/mL HRP in KRPG (Krebs-Ringer phosphate glucose) buffer was prepared (145 mM NaCl, 5.7 mM sodium phosphate, 4.86 mM KCl, 0.54 mM CaCl_2_, 1.22 mM MgSO_4_ and 5.5 mM glucose). The reaction mixture was pre-warmed in a 96-well plate with 100 μL of mixture per well. A 20 μL aliquot of cells suspended in KRPG buffer (~1.5 × 10^4^ cells) were added to each well. Samples were incubated for 1 h. Fluorescence was measured with a microplate reader (Molecular Devices; San Jose, CA, USA).

### 4.8. Lipids

Lipids were quantified by shotgun lipidomics using an ABI 5600+ (AB Sciex, Framingham, MA, USA), as described previously [39,40]. Briefly, we simultaneously identified changes in hundreds of distinct lipid species via a nonbiased approach following direct infusion of extracted lipids containing 18 mM ammonium fluoride to aid in ionization of neutral lipids and to reduce salt adducts. Data from the AB Sciex 5600+ was collected and calibrated with Analyst and PeakView Software (AB Sciex). The in-house-developed Lipid Explorer software assists with simplifying the data by identifying lipid species based on exact mass and fragmentation patterns.

### 4.9. Tissue Homogenization

Muscles were ground-glass homogenized in lysis buffer (50 mM Tris-HCl, pH 7.4; 250 mM mannitol; 50 mM NaF; 5 mM sodium pyrophosphate; 1 mM EDTA; 1 mM EGTA; 1% Triton X-100; 50 mM β-glycerophosphate; 1 mM sodium orthovanadate; 1 mM DTT; 1 mM benzamidine; 0.1 mM phenylmethane sulfonyl fluoride; 5 μg/mL soybean trypsin inhibitor). An aliquot of the homogenate was separated for the citrate synthase assay, while the rest was centrifuged at 10,000× *g* for 10 min. The supernatant was retained for western blotting analysis.

### 4.10. Citrate Synthase Activity

Whole, uncentrifuged homogenates were assayed for citrate synthase activity as described previously [41], with the exception that reaction volumes were scaled down to a final volume of 200 μL to be run in 96-well plates.

### 4.11. Western Blotting

Muscle homogenates were analyzed for protein concentration using a modified Lowry assay (DC Protein Assay; Bio-Rad Laboratories, Hercules, CA, USA) according to manufacturer’s protocols. Equal amounts of protein were then separated by electrophoresis and transferred to polyvinylidene fluoride (PVDF) membranes. Proper transfer and equal protein loading were verified by Ponceau-S staining of the PVDF membranes after transfer. Primary antibodies [OXPHOS (Invitrogen #457999), cytochrome C (Santa Cruz #sc-13156), MFN2 (EMD-Millipore #ABC42), DRP1 (Cell Signaling, #8570)] were applied overnight at 4 °C. Membranes were exposed to autoradiographic film and resulting band intensities were determined using Gel-Pro software (Media Cybernetics, Rockville, MD, USA).

### 4.12. Statistical Methods

Data are presented as means ± SEM. Data were compared with Student’s *t*-test (Graphpad Prism; Microsoft Excel). Significance was set at *p* < 0.05.
